# Supplementary material for: Gre factors help Salmonella adapt to oxidative stress by improving transcription elongation and fidelity of metabolic genes
Source: PLoS Biol. 2023 Apr 4;21(4):e3002051. doi: 10.1371/journal.pbio.3002051 (PMC10072461; doi:10.1371/journal.pbio.3002051)
Supplement: S1 Text — Table a in S1 Text. Bacteria used in this study. Table b in S1 Text. Plasmids used in this study. Table c in S1 Text. Oligonucleotides used in this study. Fig a in S1 Text. KEGG pathway overrepresentation analysis of SNSs. Fig b in S1 Text. Effect of carbon source on Salmonella growth. Fig c in S1 Text. Amino acid pools in Salmonella grown on glucose. Fig d in S1 Text. RNA seq analysis of Salmonella grown in glucose. Fig e in S1 Text. Resolution of transcriptional pausing by proteins that bind to the secondary channel of RNA polymerase. Fig f in S1 Text. Susceptibility of aerobic respiration mutants to peroxide stress. (DOCX) [file pbio.3002051.s003.docx]

Supplementary Materials

Gre factors help *Salmonella* adapt to oxidative stress by improving transcription elongation and fidelity of metabolic genes

Sashi Kant^1^, James Karl A Till^1^, Lin Liu^1^, Ju-Sim Kim^1^, and Andres Vazquez-Torres^1,2,*^

^1^University of Colorado School of Medicine, Department of Immunology & Microbiology, Aurora, Colorado, USA

^2^Veterans Affairs Eastern Colorado Health Care System, Denver, Colorado, USA

^*^Corresponding Author: Andrés Vázquez-Torres

E-mail: [Andres.Vazquez-Torres@cuanschutz.edu](mailto:Andres.Vazquez-Torres@cuanschutz.edu),

**Table a. Bacteria used in this study.**

**Strains Relevant characteristics Reference**

***Salmonella***

14028s wild type of *S. enterica* serovar *Typhimurium* ATCC

AV18260 Δ*greA*::Chl [Δ*greA*] This study

AV18261 Δ*greB*::Km [Δ*greB*] This study

AV19001 Δ*greAB*::Chl [Δ*greA*Δ*greB*] This study

AV20059 Δ*greAB*::Chl [pWSK29::*greA*] This study

AV20060 Δ*greAB*::Chl [pWSK29::*greB*] This study

AV0660 Δ*relA*::Km [Δ*relA*] ^1^

AV0436 Δ*nuo*Δ*ndh*::Km [Δ*nuo*Δ*ndh*] ^2^

AV21184 Δ*mgsA*::Km [Δ*mgsA*] This study

AV21185 Δ*mgsA*Δ*greAB*::Km:Chl [Δ*mgsA*Δ*greA*Δ*greB*] This study

AV21186 Δ*gloA*::Km [Δ*gloA*] This study

AV21187 Δ*gloA*Δ*greAB*::Km:Chl [Δ*gloA*Δ*greA*Δ*greB*] This study

AV21031 Δ*gloB*::Km [Δ*gloB*] This study

AV21043 Δ*gloB*::Pn [Δ*gloB*] This study

AV21044 Δ*gloB*Δ*greAB*::Km:Chl [Δ*gloB*Δ*greA*Δ*greB*] This study

AV09214 Δ*katG*::Km [Δ*katG*] ^3^

AV21196 WT [pfpv25::*roGFP2*] This study

AV21197 Δ*greAB*::Chl [pfpv25::*roGFP2*] This study

***E.coli***

DH5α *supE*44 Δ*lacU*169 (φ80 *lacZ* ΔM15) *hsdR*17 *recA*1 ^4^ *endA*1 *gyrA*96 *thi-*1 *relA*1

BL21(DE3) F^-^ *ompT hsd*S_B_(r_B_^-^ m_B_^-^) *gal* *dcm* (DE3) Invitrogen

AV21188 BL21(DE3) (pET14b::*greA*) This study

AV21189 BL21(DE3) (pET14b::*greB*) This study

AV21190 DH5α (pWSK29::*greA*) This study

AV21191 DH5α (pWSK29::*greB*) This study

AV10267 BL21(DE3) (pGEX-6P-1::*dksA*) ^5^

AV21193 DH5α (pTIM::*cydA1*) This study

AV21194 DH5α (pTIM::*cydA2*) This study

AV21195 DH5α (pTIM::*gapA*) This study

AV22039 DH5α (pTIM::*eno*) This study

* Strains names briefly described in the text are indicated by square brackets ([ ]) next to their full names.

**Table b. Plasmids used in this study.**

**Plasmid Relevant characteristics Source**

pET14b *ori* pBR322, N-terminal 6His·Tag Novagen

expression vector, Pn^r^

pTIM *in vitro* transcription backbone plasmid, ^6^

bla *rrnB* & *rpoC* term pBluescript, Pn^r^

proGFP2 pfpv25-roGFP2 ^7^

pWSK29 low copy plasmid, *lacZα*, Pn^r^  ^8^

pET14b::*greA* pET14b + 477 bp *greA* CDS, Pn^r^ This study

pET14b::*greB* pET14b + 474 bp *greB* CDS, Pn^r^ This study

pTIM::*cydA* 1 pTIM *+* 300 bp p*cydA* and 50 bp *cydA* CDS, Pn^r^ This study

pTIM::*cydA* 2 pTIM *+* 300 bp p*cydA* and 400 bp *cydA* CDS, Pn^r^ This study

pTIM::*gapA* pTIM *+* 300 bp p*gapA* and 50 bp *gapA* CDS, Pn^r^ This study

pTIM::*eno* pTIM *+* 300 bp p*eno* and 50 bp *eno* CDS, Pn^r^ This study

pWSK29::*greA* pWSK29 + 877 bp *greA* CDS with promoter, Pn^r^ This study pWSK29::*greB* pWSK29 + 874 bp *greB* CDS with promoter, Pn^r^ This study

**Table c. Oligonucleotides used in this study.**

| **Strains** | **Primer Sequence (5’ → 3’)** |
| --- | --- |
| Δ*greA*::Cm  Δ*greB*::Km  Δ*mgsA*::Km  Δ*gloA*::Km  Δ*gloB*::Km | **F:**CATTGCCCCCTACAGGAATGTTCAAGAGGTATAACAAATGGTGTAGGCTGGAGCTGCTTC  **R:**TTTACAATACACCAACAATTTGCGTATTGAGTACTGCTTACATATGAATATCCTCCTT |
|  | **F:**GTGTGCGCAATATCGACAGCAAAGGTAAATCAACGAGATGGTGTAGGCTGGAGCTGCTTC  **R:**TGCCAGCCATCAGCGGGGGCTTAGGATTCTTCTTGTCTTAATTCCGGGGATCCGTCGACC |
|  | **F:**ATGGAACTGACGACTCGCACCTTGCCGACGCGCAAACATACGGCAAACAAACCACCGCTG  **R:**TTATTTCAGGCGCTCGGCCAGATAACGCGCATAATCCGGATTAGAAAAACTCATCGAGCA |
|  | **F:**ATGCGTTTATTGCATACTATGCTGCGCGTCGGCGATTTGCCGGCAAACAAACCACCGCTG  **R:**TCAGTTACCCAGACCGCGGCCTGCGTCTTTAGCTTCAATCTTAGAAAAACTCATCGAGCA |
|  | **F:**ATGAATCTTAACAGTATTCCCGCGTTTCAGGACAATTACACGGCAAACAAACCACCGCTG  **R:**TCAGAACGTGTCTTTCTTTGACCTTAACCATGCAAAACGCTTAGAAAAACTCATCGAGCA |
| **Plasmid** |  |
| pET14b::*greA* | **F:**  AAA *CAT ATG* CAA GCT ATT CCG ATG ACC TTA |
|  | **R:** AAA *GGA TCC* TTA AAG GTA TTC CAC TTT AAG CAC |
| pET14b::*greB* | **F:** AAA *CAT ATG* AAA ACG CCC CTG ATC ACC  **R:** AAA *GGA TCC* TTA TTT GAC GTA TTC GAT CGC |
| pTIM::*cydA1/2* | **F:** AAA *GAA TTC* AAA TTG TCC GTG ATC AAA  **R1:**AAA *AAG CTT* ATC GCG GTC AAG GCA AAC  **R2:** AAA *AAG CTT* CCA CCA GCC AGG TGA CGC |
| pTIM::*gapA* | **F:** AAA *GAA TTC* GAT TCT AAC AAA ACA TTA ACA  **R:** AAA *AAG CTT* ACA ATG CGA CCG ATA CGG |
| pTIM::*eno* | **F:** AAA *GAA TTC* ACA ATA TGT TGT TGA AAC AAA  **R:** AAA *AAG CTT* CCA CGG GAG TCG ATG ATT |
| pWSK29::*greA* | **F:** AAA *CCG CGG* ATC AAT AGC CGG CGC  **R:** AAA *GGA TCC* TTA AAG GTA TTC CAC TTT AAG CAC |
| pWSK29::*greB* | **F:** AAA *CCG CGG* GCA GCA TTA AAT CCA GTA  **R:**  AAA *GGA TCC* TTA TTT GAC GTA TTC GAT CGC |
| **qRT-PCR** |  |
| *greA* | **F:** CATCGTCGCCAACAATGCGG  **R:** AGCAGGGTTTCTGCGAAGGG |
| *greB* | **F:** AAAGTGACCTGGGCTGCGAG  **R:** AGACTTTGCCCTCCTGCTGC |
| *dksA* | **F:** CGACCGGATCGGGGAAGTTG  **R:** TACCAGGAGAAACCGGGCGA |
| *cydA* | **F:** GTTTGTGCCGCTAACGCTCG  **R:** CGCACCGAAGATGTCCCCAA |
| *frdA* | **F:** GCGTCGCTTCGGTGGTATGA  **R:** CACCAGCGTGCCTTCCATCA |
| *cyoA* | **F:** CGCTGCTGGATCCCAAAGGA  **R:** GTCCAGACCACGGCTTCCAC |
| *atpI* | **F:** TCTGGGGCATCTCCGCAGTA  **R:** ATCAGCGGCAAAAACACCGC |
| *atpG* | **F:** GAGATGGTCGCCGCTTCCAA  **R:** CAAGCCACCGCACAAACCAC |
| *atpF* | **F:** AACGCAACAATCCTCGGCCA  **R:** CCTGAGCTTCCGCTTTCGCT |
| *atpE* | **F:** TCTGCTGTACATGGCTGCCG  **R:** ACGCGACAGCGAACATCACG |
| *nuoM* | **F:** AGCGCTGATTACCATGGGGC  **R:** TCTCGCCAGGAGCAAAGCAC |
| *nuoG* | **F:** ATACGAGGTCAATGGCGCGG  **R:** CAGGCGACCACGCGTATCTT |
| *nuoC* | **F:** TTTACCGTTCAGGCGACCCG  **R:** ACGCGCAGGTCGTTTTCAGA |
| *nuoA* | **F:** CTGTGCTGCCTGATGCTGGT  **R:** CAGCCGCTTTCGCGGATAGA |
| *mgsA* | **F:** ACTGGGTGGAACGCCATCAG  **R:** AATGAGTGCGCCAACCTGCT |
| *gloA* | **F:** GGGGCGTTGAGAGCTACGAC  **R:** CTTTTACCGGCCCCGCTTCA |
| *gloB* | **F:** CGGCGGAAACGCAAGACAAG  **R:** AGCGTGTCGCCGCAGAATAA |
| *dgaF* | **F:** TACTCACCCGGCCCATGTCA  **R:** GTACCGCCGCTTCACAGGAA |
| *talA* | **F:** CGACATCGAGTCCATCCGCC  **R:** CGCGCCAAAATTAACCGCCA |
| *deoC* | **F:** TCCGCATTGCAACGGTGACT  **R:** CAATACGTTCGCTGCGGCAC |
| *sucC* | **F:** TTTCCTTGAGCGCGACCTGG  **R:** CCATGCAACCGATGTTGCCG |
| *acnA* | **F:** GGCATTCAGCCGTTTCAGCG  **R:** TGGGAGTCAGTCCCCACCAG |
| *STM14_0886* | **F:** TGTCCGTCGTGTAGGAGGCA  **R:** CGCCCATACCTAAAGGGCCG |
| *aceF* | **F:** ATCGAAACTCGCGACTGGCT  **R:** ATGCGACGTTCCAGCTCCAG |
| *pykF* | **F:** CCGACAAATCCGTGGTCGGT  **R:** AACGCCTTTGTTCTCGCCCA |
| *eno* | **F:** CTGCGCGATGGCGACAAATC  **R:** CCAGAATGGCGTTTGCACCG |
| *gapA* | **F:** GCTGGTGGAATATATGACT  **R:** ACAATGCGACCGATAC  **Probe:** CCAAAACCGTTGATACCTACT |

* Restriction enzyme sites are italics.

**Supplementary figure**

**
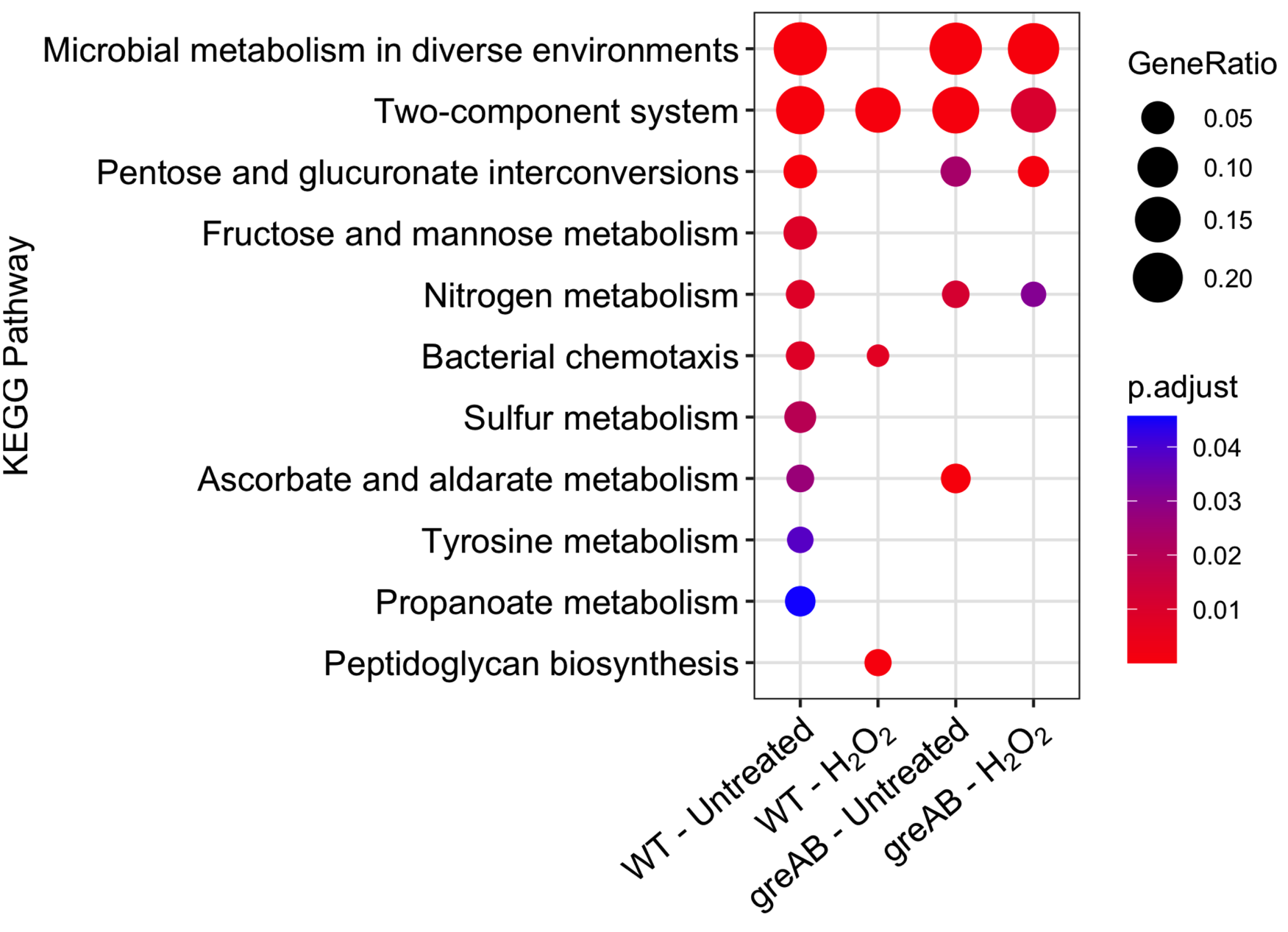
**

**Fig a in S1 Text. KEGG pathway over-representation analysis of SNSs**. The gene ratio per pathway term, which is the ratio of input genes that are annotated in that term to the total number of annotated genes in the sample, is represented by dot size and the adjusted *p*-value of the enriched term by dot color. Data are the mean (N=4).

**
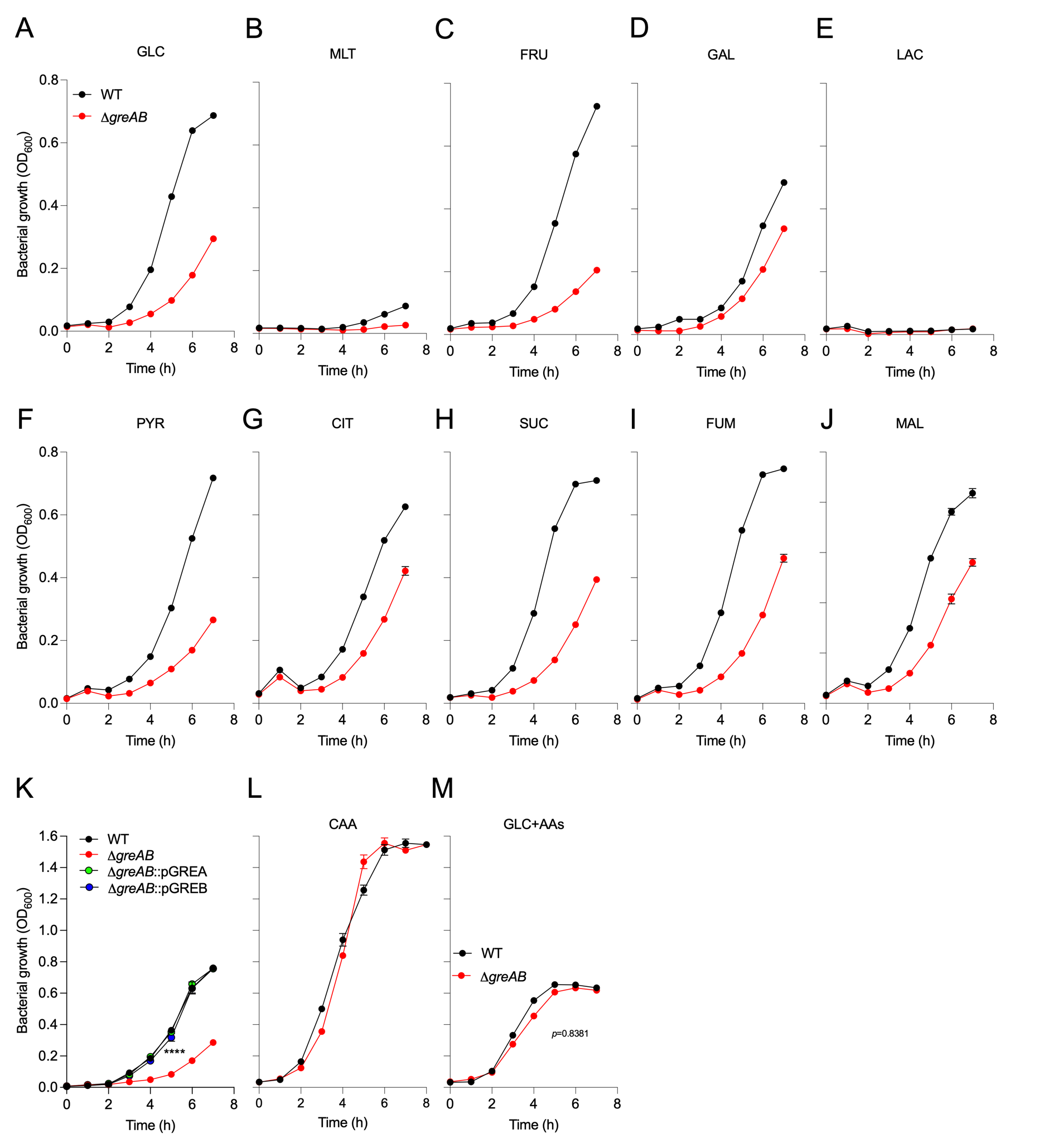
**

**Fig b in S1 Text. Effect of carbon source on *Salmonella* growth.** Growth of wild-type (WT) and Δ*greAB* *Salmonella* in MOPS minimal medium, pH 7.2, at 37⁰C in a shaker incubator as measured by OD_600_ (A, K). Where indicated, MOPS was supplemented with glucose (A, K), maltose (B), fructose (C), galactose (D), and lactose (E), pyruvate (F), citrate (G), succinate (H), fumarate (I), malate (J), Casamino acids (L), or glucose and all 20 amino acids (M). Data are shown as mean ± S.D. (N=3). GLC, Glucose; MLT, Maltose; FRU, Fructose; GAL, Galactose; LAC, Lactose; PYR, Pyruvate; CIT, Citrate; SUC, Succinate; FUM, Fumarate; MAL, Malate; Casamino acids (CAA); 40 μg/ml each of all 20 amino acids (AAs).

**
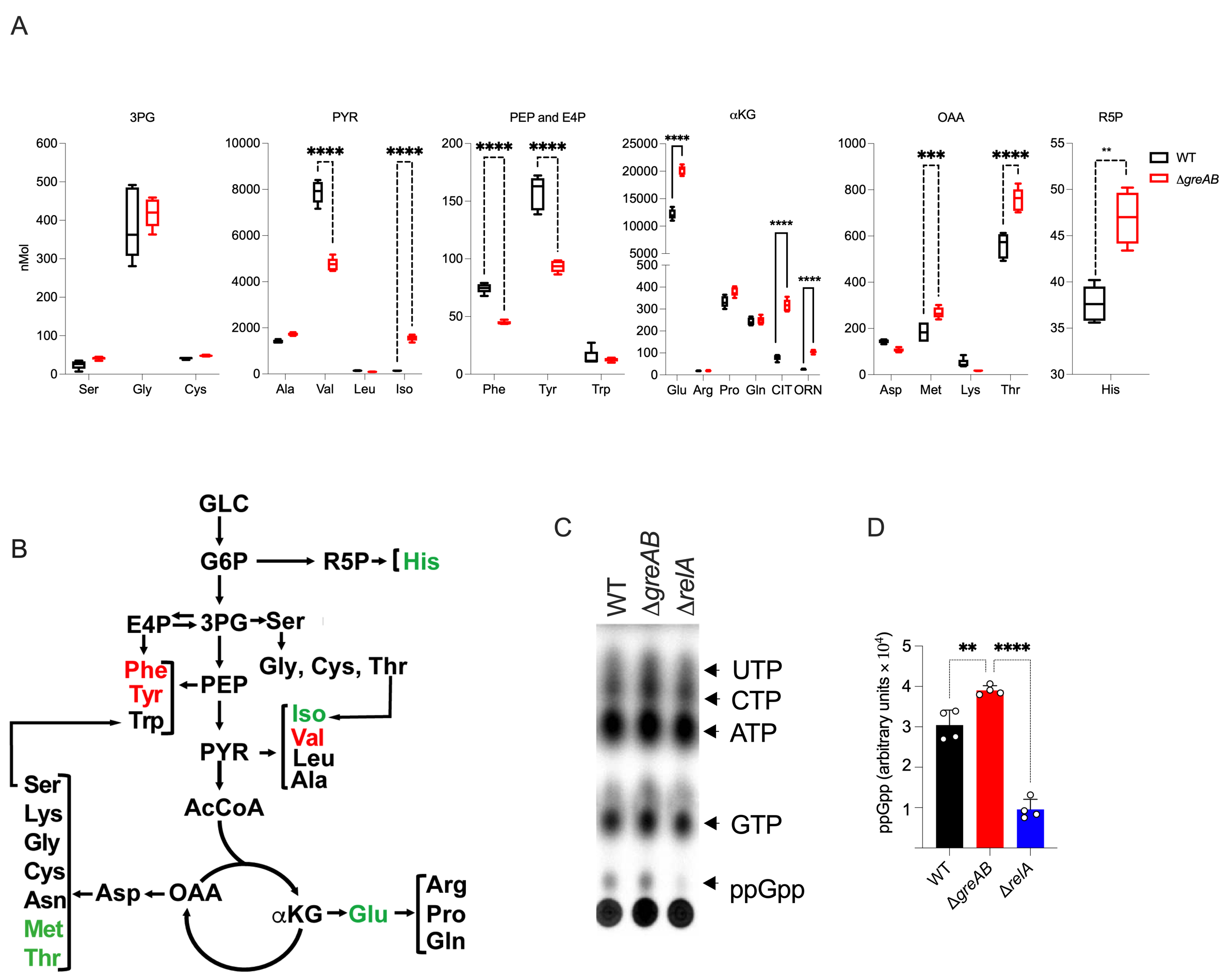
**

**Fig c in S1 Text.** **Amino acid pools in *Salmonella* grown on glucose.** (A) Amino acids were quantified in *Salmonella* grown in MOPS-GLC media, pH 7.2 at 37⁰C to an OD_600_ of 0.25 by liquid chromatography mass spectrometry (LC-MS). Data are the mean ± S.D (N=5). ***, ****; *p*< 0.001 and *p*< 0.0001, respectively, as determined by two-way ANOVA. Three letter amino acid code is used. 3PG, phosphoglycerate; PYR, pyruvate; PEP, phosphoenolpyruvate; E4P, erythrose 4-phosphate; αKG, alpha-ketoglutarate; OAA, oxaloacetic Acid; R5P, ribose 5-phosphate; CIT, citrulline; ORN, ornithine.(B) Amino acids that were statistically (*p*<0.01) more and less abundant in Δ*greAB*than WT controls are shown in green and red, respectively. AAs contained at similar levels in Δ*greAB*and WT *Salmonella*are shown in black. Data are the mean ± S.D. (N=5). (C, D) Analysis for nucleotide biosynthesis. TLC autoradiogram (C) and densitometry (D) of ^32^P-labeled nucleotides extracted from an OD_600_ of 0.25 of *Salmonella* strains grown in MOPS-GLC media, pH 7.2. Data are shown as mean ± S.D. (N=4). **,**** *p*<0.01 and *p*<0.0001, respectively, as determined by one-way ANOVA.

**
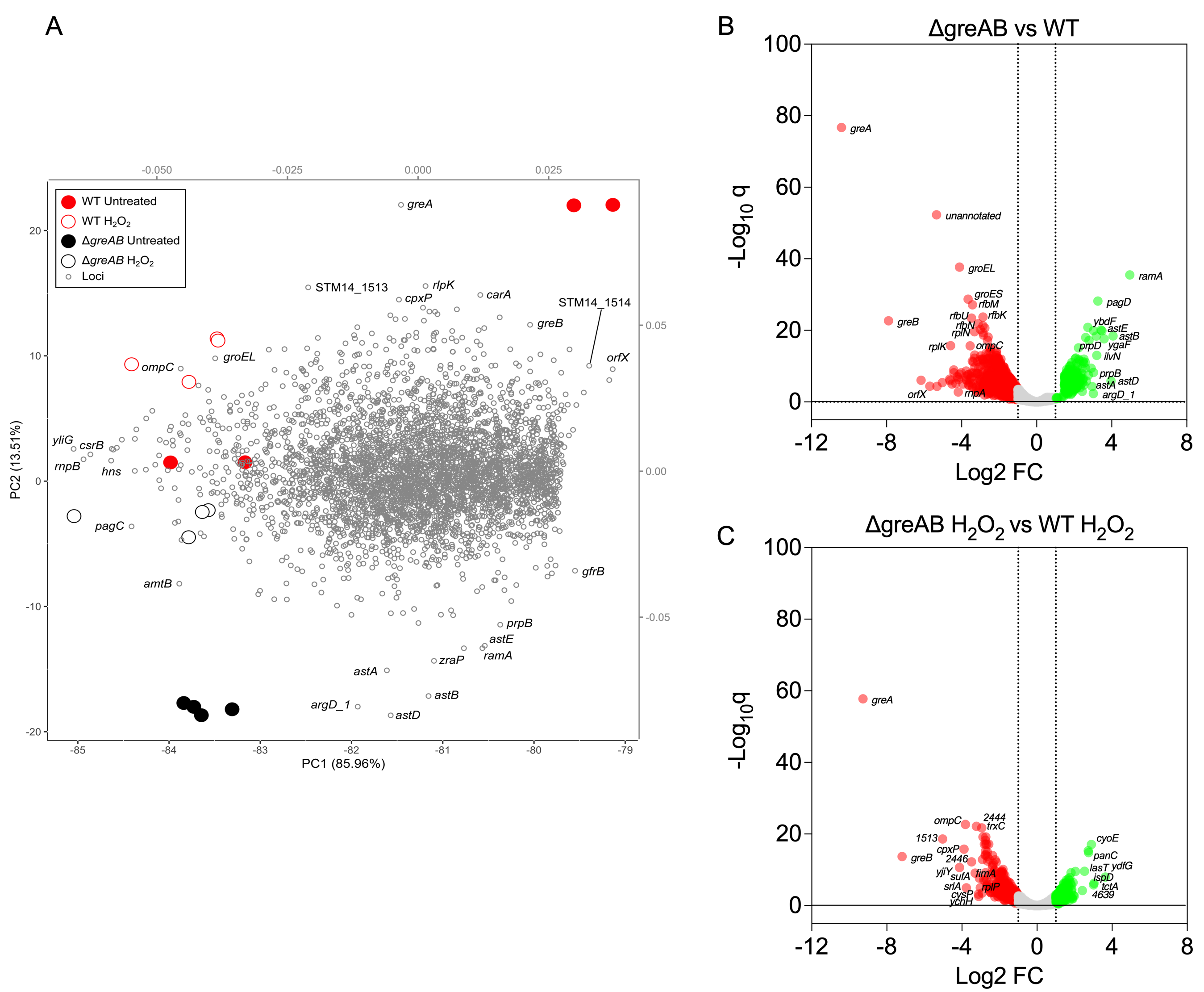
**

**Fig d in S1 Text. RNA seq analysis of *Salmonella* grown in glucose.** (A) A principal component analysis of RNAseq data obtained from WT and ∆*greAB* *Salmonella* grown in MOPS-GLC minimal medium, pH 7.2, at 37ºC to OD_600_ of 0.25. Where indicated, the bacteria were treated with 400 μM H_2_O_2_ for 30 min. The PCA was performed in R on log-transformed and Pareto-scaled counts from each loci. Loadings are shown by the small gray points and with the gray axes. (B, C) Volcano plot and q-value distributions for comparisons of RNA-seq count data after analysis with DESeq2 and edgeR with tagwise dispersion and fdr-corrected p-values. Data points are labeled where the meet the fold-change and q-value cutoff criteria stated at the bottom of each figure.

**
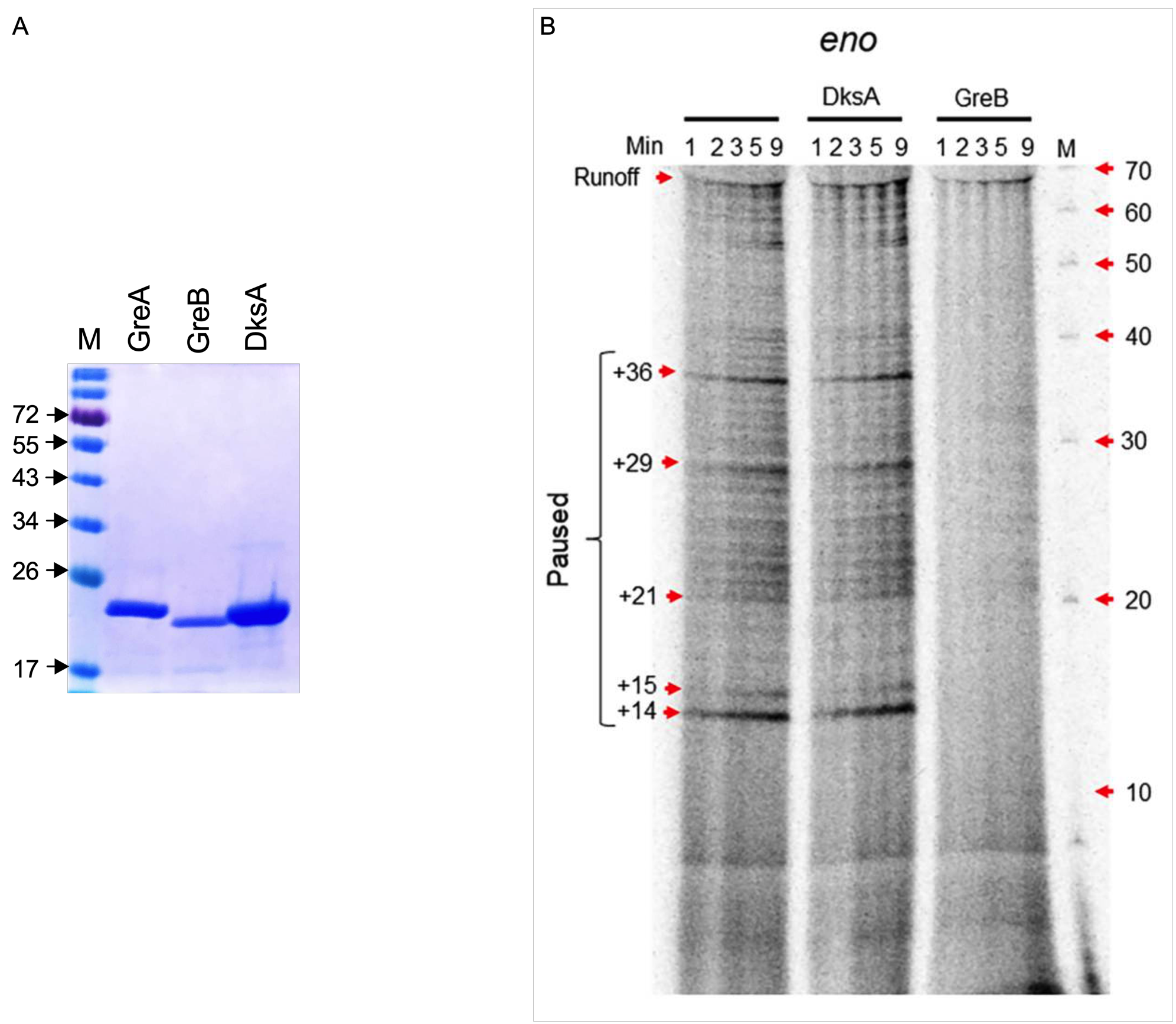
 Fig e in S1 Text. Resolution of transcriptional pausing by proteins that bind to the secondary channel of RNA polymerase.** (A) Purified proteins GreA-His, GreB-His and DksA-His were evaluated by SDS-PAGE gels and visualized by Coomassie Brilliant Blue staining. (B) Transcriptional pausing of *in vitro* transcription reactions containing *eno* templates. Where indicated, the reactions contained 1 μM DksA or 100 nM GreB recombinant proteins. The α^32^P-UTP-labaled products of the *in vitro* transcription reactions were visualized in urea PAGE gels.

**
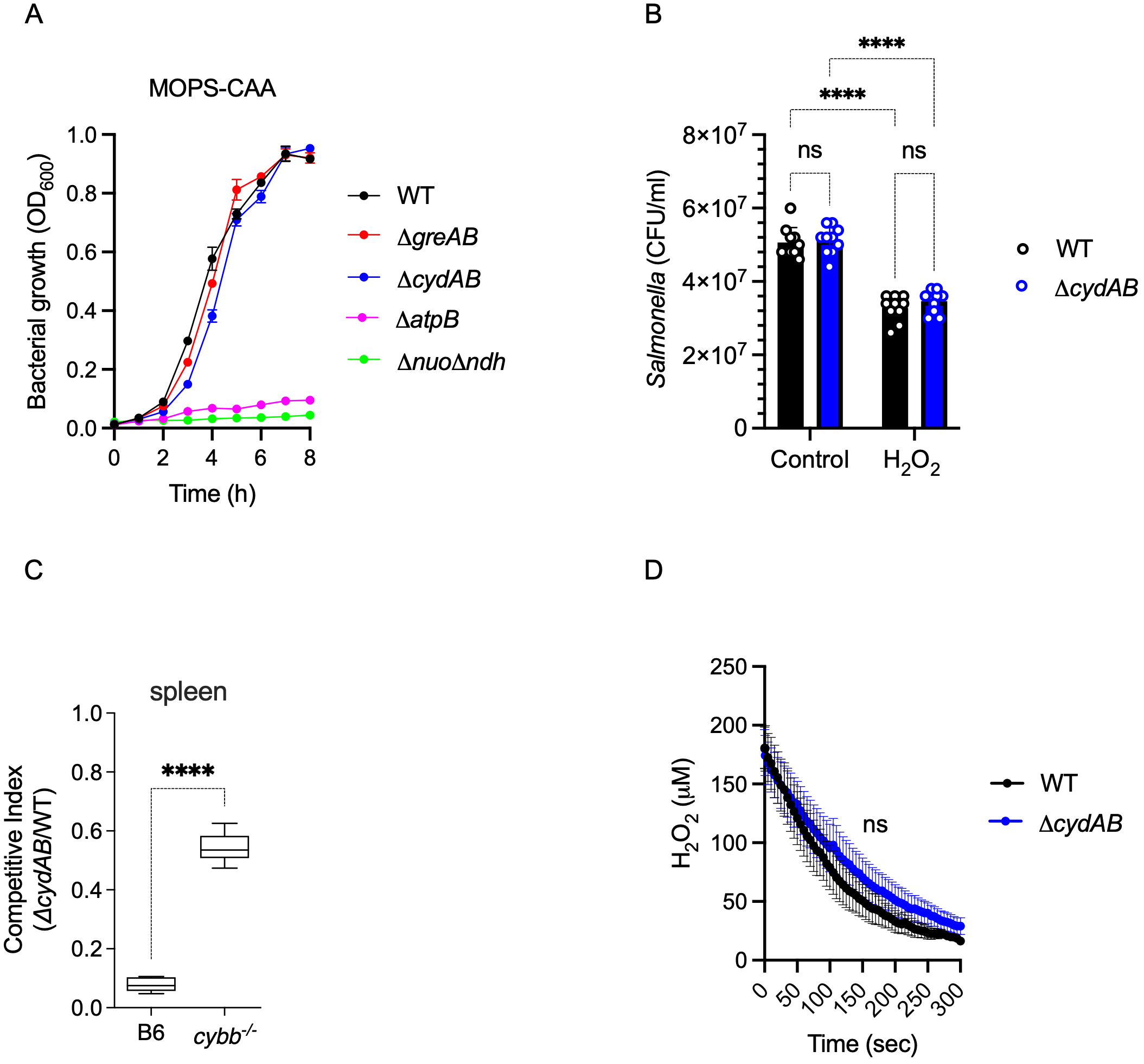
**

**Fig f in S1 Text. Susceptibility of aerobic respiration mutants to peroxide stress**. (A) Growth of *Salmonella* in MOPS-CAA minimum medium, pH 7.2. (B) H_2_O_2_killing of *Salmonella*. Bacterial cultures grown overnight in LB broth and diluted to 2×10^5^CFU/ml in PBS were treated for 2h with 200 μM H_2_O_2_. Killing is expressed as percent survival compared to bacterial counts at time zero. Data are the mean ± S.D. (N=10). ****, *p*<0.0001 as determined by two-way ANOVA. (C) Competitive index of *Salmonella* in spleen of C57BL/6 and *cybb*^-/-^mice 3 days after i.p. inoculation with 100 CFU of equal numbers of WT and Δ*cydAB* *Salmonella* (n = 10). ****, *p*<0.0001 as determined by unpaired *t*-test. (D) Degradation of H_2_O_2_ by WT and Δ*cydAB* *Salmonella* grown in MOPS-GLC media, pH 7.2 at 37⁰C to an OD_600_ of 0.25 as measured polarographycally in an ISO-OXY/HPO analyzer equipped with a H_2_O_2_ probe. 200 μM H_2_O_2_ were added to the cultures immediately before measurements were initiated. Data are the mean ± S.D. (N=6). *p*< 0.1116 as determined by unpaired *t*-test.

**REFERENCES**

1 Fitzsimmons, L. F., Liu, L., Kim, J. S., Jones-Carson, J. & Vazquez-Torres, A. Salmonella Reprograms Nucleotide Metabolism in Its Adaptation to Nitrosative Stress. *mBio* **9**, doi:10.1128/mBio.00211-18 (2018).

2 Husain, M. *et al.* Nitric oxide evokes an adaptive response to oxidative stress by arresting respiration. *J Biol Chem* **283**, 7682-7689, doi:M708845200 [pii]

10.1074/jbc.M708845200 (2008).

3 Chakraborty, S. *et al.* Glycolytic reprograming in Salmonella counters NOX2-mediated dissipation of DeltapH. *Nat Commun* **11**, 1783, doi:10.1038/s41467-020-15604-2 (2020).

4 Hanahan, D. Studies on transformation of Escherichia coli with plasmids. *J Mol Biol* **166**, 557-580, doi:10.1016/s0022-2836(83)80284-8 (1983).

5 Kim, J. S. *et al.* DksA-DnaJ redox interactions provide a signal for the activation of bacterial RNA polymerase. *Proc Natl Acad Sci U S A* **115**, E11780-E11789, doi:10.1073/pnas.1813572115 (2018).

6 Tapscott, T. *et al.* Guanosine tetraphosphate relieves the negative regulation of Salmonella pathogenicity island-2 gene transcription exerted by the AT-rich ssrA discriminator region. *Sci Rep* **8**, 9465, doi:10.1038/s41598-018-27780-9 (2018).

7 van der Heijden, J. *et al.* Exploring the redox balance inside gram-negative bacteria with redox-sensitive GFP. *Free Radic Biol Med* **91**, 34-44, doi:10.1016/j.freeradbiomed.2015.11.029 (2016).

8 Wang, R. F. & Kushner, S. R. Construction of versatile low-copy-number vectors for cloning, sequencing and gene expression in Escherichia coli. *Gene* **100**, 195-199 (1991).
