# Supplementary figures and images for: Gre factors help Salmonella adapt to oxidative stress by improving transcription elongation and fidelity of metabolic genes
Source: PLoS Biol. 2023 Apr 4;21(4):e3002051. doi: 10.1371/journal.pbio.3002051 (PMC10072461; doi:10.1371/journal.pbio.3002051)

Fig. 4B

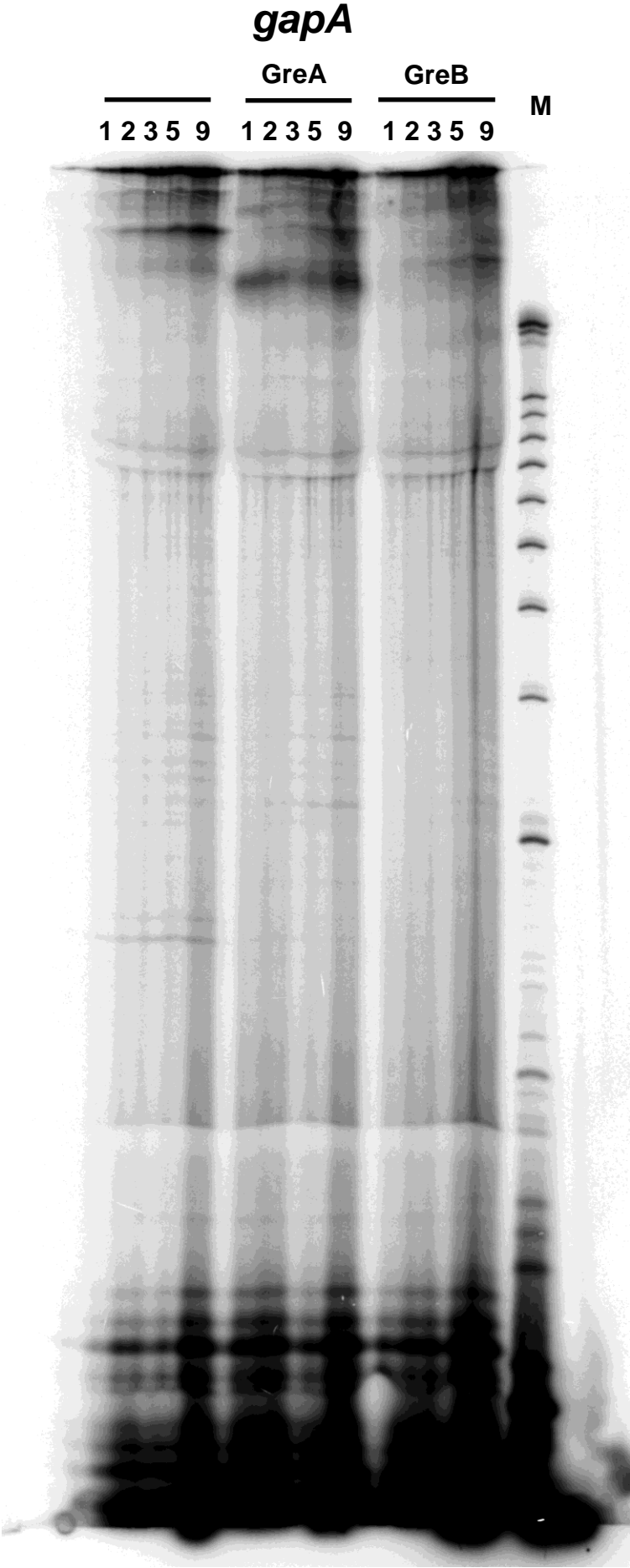

**Fig 4C**

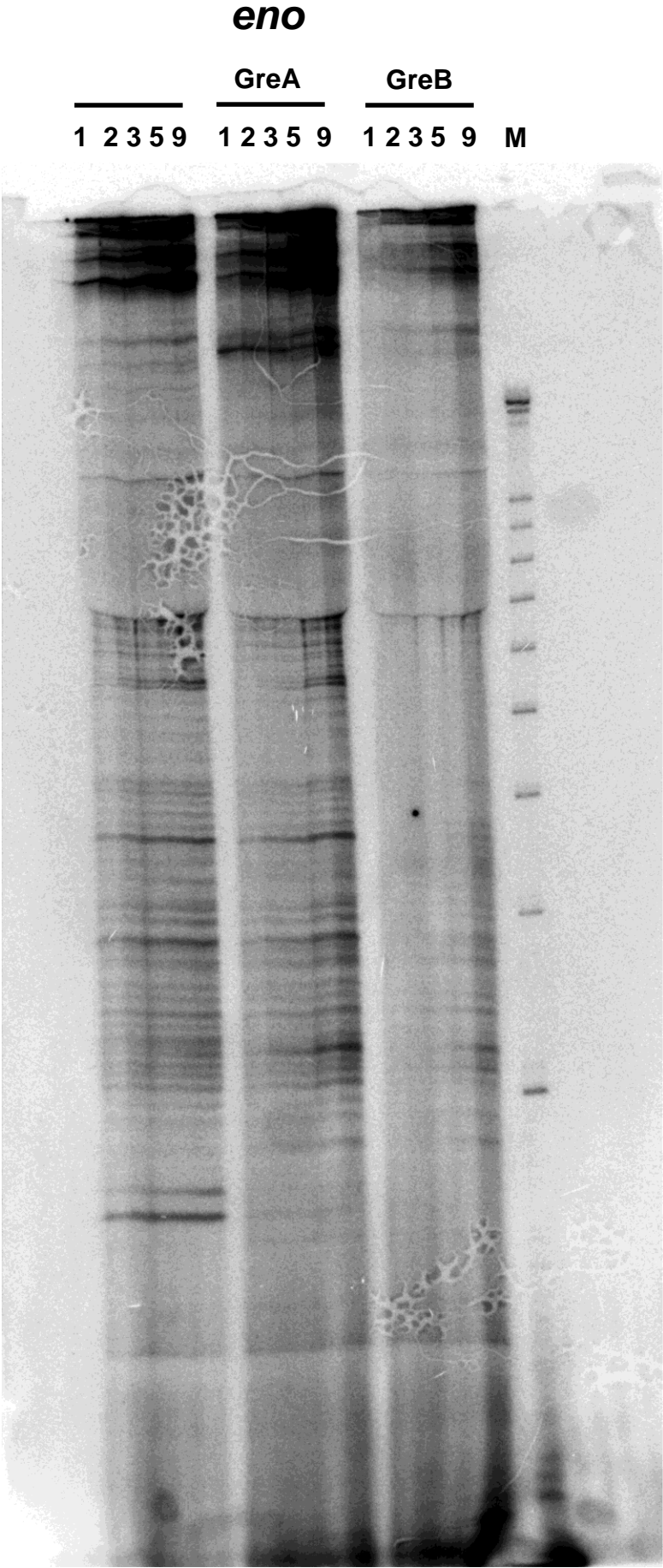

**Fig 4D**

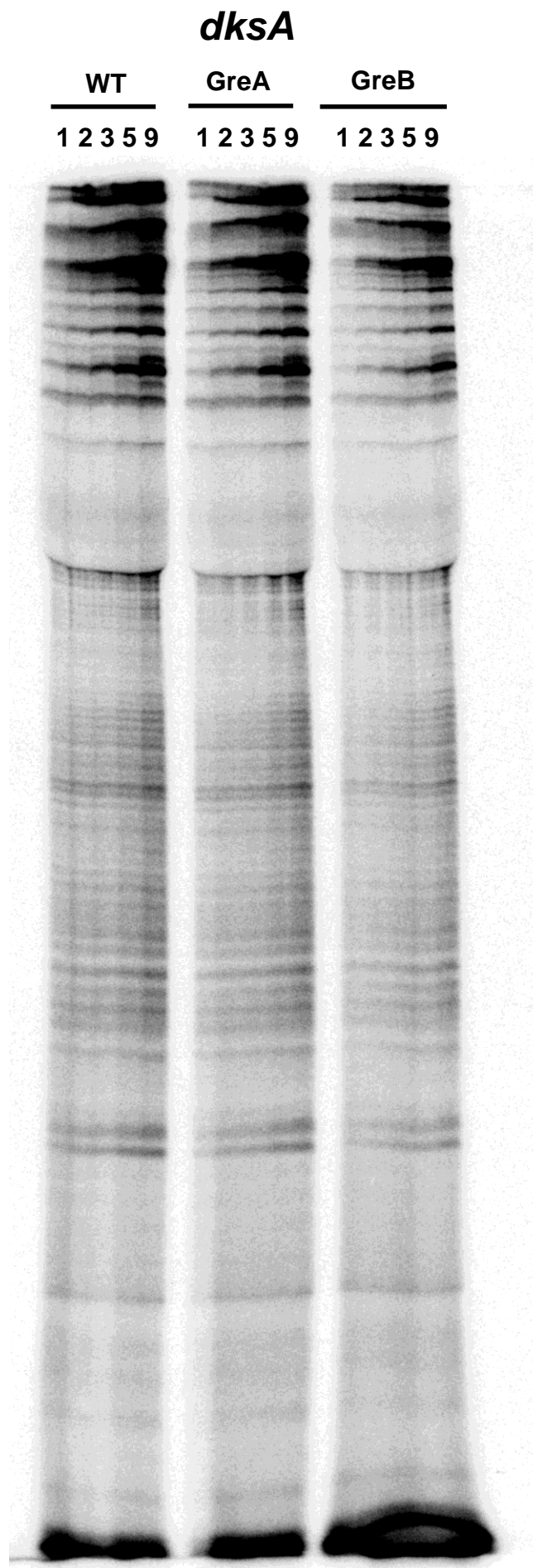

Fig 5B

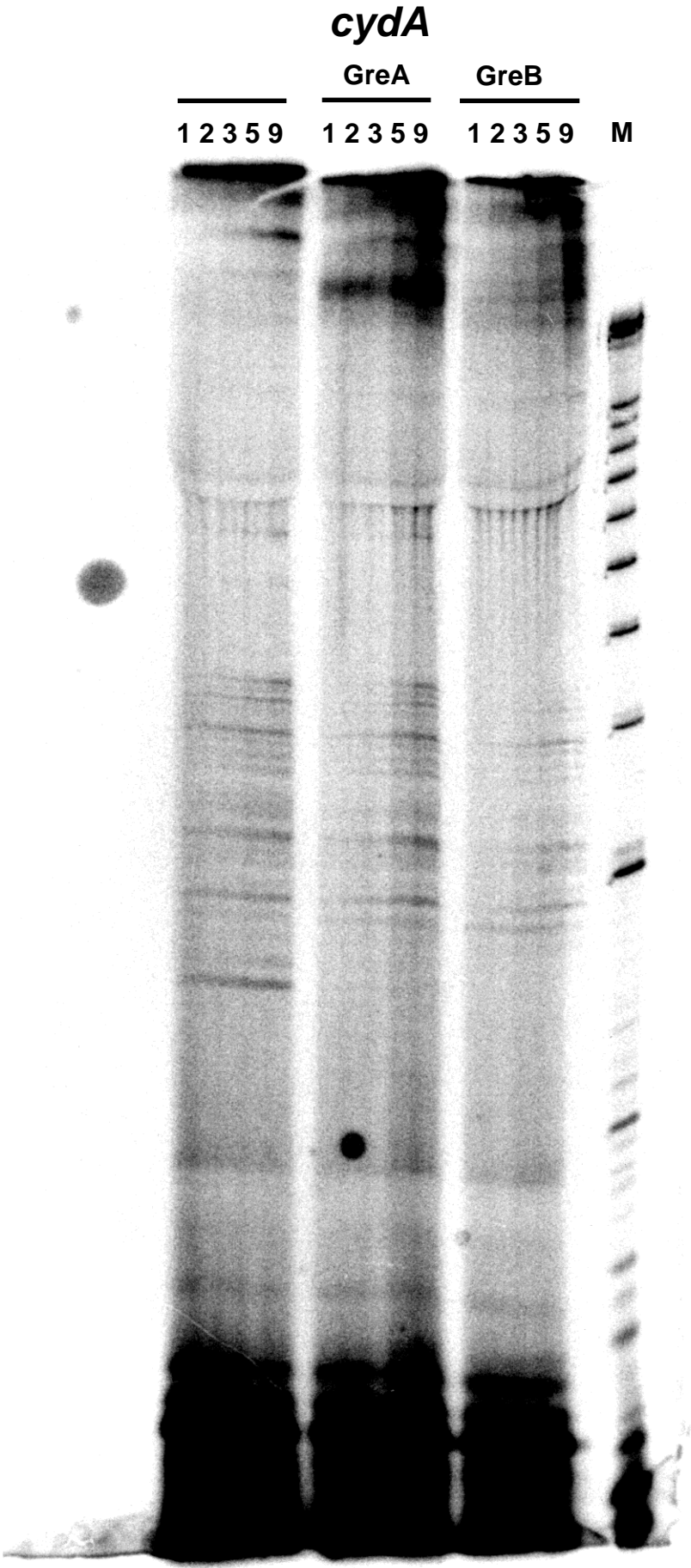

Fig. S3C

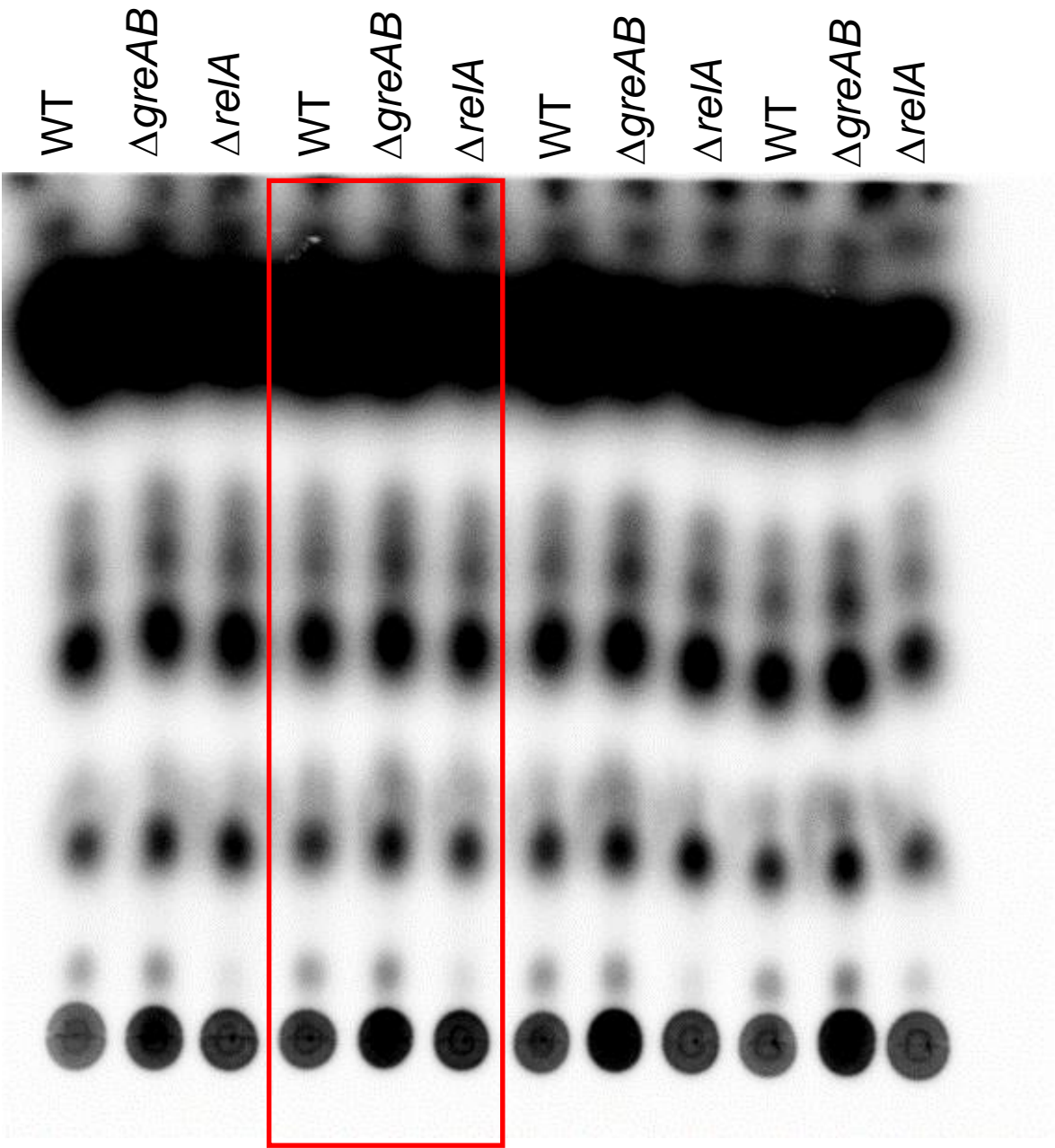

**Fig. S5A**

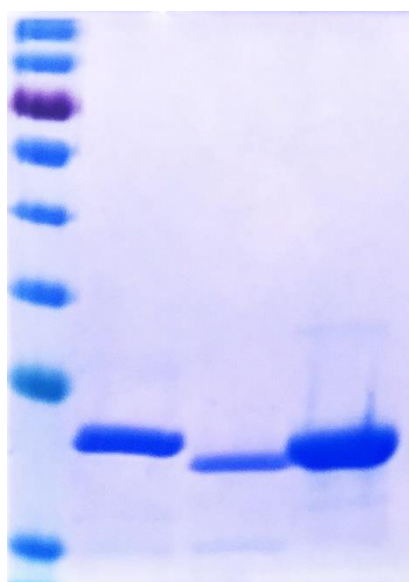

**Fig. S5B**

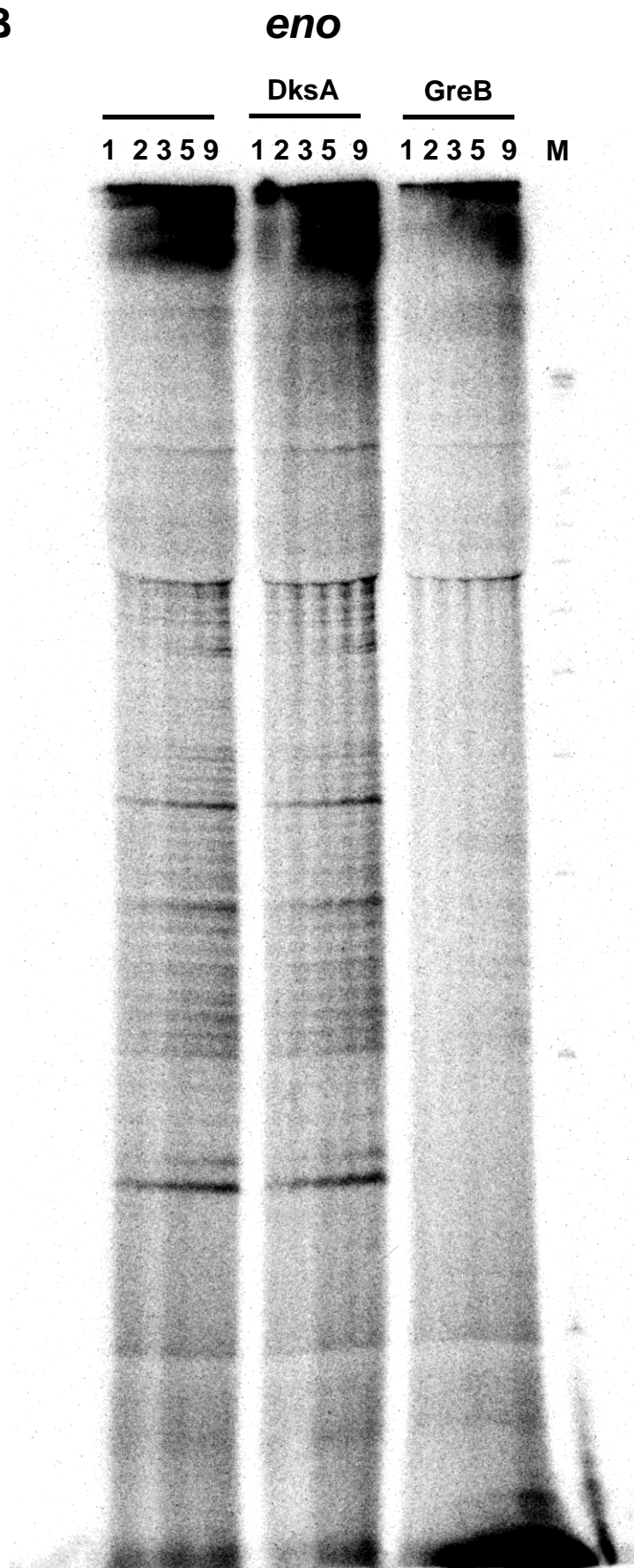

Supplement: S1 Raw Images — (PDF) [file pbio.3002051.s005.pdf]
