# Supplementary material for: Gre factors help Salmonella adapt to oxidative stress by improving transcription elongation and fidelity of metabolic genes
Source: PLoS Biol. 2023 Apr 4;21(4):e3002051. doi: 10.1371/journal.pbio.3002051 (PMC10072461; doi:10.1371/journal.pbio.3002051)
Supplement: Attachment — Submitted filename: Response.docx [file pbio.3002051.s006.docx]

Responses to Reviewer #1:

In this study, Kant et al. revealed a novel transcriptional mechanism by which Salmonella reprograms its metabolism for protection against oxidative stress. This mechanism is mediated by the GreA and GreB proteins, which resolve transcriptional pausing at genes for EMP (Embden-Meyerhof-Parnas) glycolysis and for ETC (Electron Transport Chain).  The authors demonstrate that this regulation protects Salmonella from the cytotoxicity of phagocyte NADPH oxidase in the innate host response. The findings reported by Kant et al. show a role for GreA and GreB transcriptional elongation factors in promoting bacterial growth in a specific stress condition. The manuscript is excellently written, technically sound, conclusions are fully supported by data, and findings are highly relevant in the biology field.
Response: Thank you for your support.

The Figures are blurred.

Response: The figures were generated in Prism and saved as tiff files at a resolution of 300 dpi in Photoshop. We are not sure why the quality of the figures was so poor. We have flattened the images in Photoshop. Hopefully, this will improve the quality of the figures.

Responses to Reviewer #2:

First I must sincerely apologize to the authors, reviewers and editors for being several days late on this review. The paper by Kant et al. explores the roles of the GreA and GreB factors in the adaptation of Salmonella to oxidative stress.  It has a lot of very intriguing data and some important insights.  I'm excited by the general trajectory of the hypothesis and its novelty.

Response: Thank you for finding the general trajectory of the hypothesis novel.

The paper never really comes full circle to answer the following important questions: `are GreAB really controlling the expression of metabolic genes (directly) under oxidative stress, or is this just a coincidental defect that occurs in a highly pleiotropic set of mutants? The general thrust of the narrative is that GreAB somehow fix pausing during oxidative stress but whether this pausing occurs at higher rates during oxidative stress remains unclear (if I missed some important data I must apologize...I just can't figure out where this might have been addressed).

Response: It is unlikely that Gre factors directly respond to oxidative stress. The globular domain of Gre factors lacks the cysteine residues that have been shown to help DksA sense oxidative and nitrosative stress. We did not do *in vitro* transcription or pausing experiments under oxidative stress conditions, because Gre factors control elongation and fidelity after RNA polymerase has already been recruited by transcription factors to target promoters. Our principal component analysis shows that the transcriptional responses of wildtype and Δ*greAB* *Salmonella* trend to converge following H_2_O_2_ treatment (**Fig. S4A**). The selective activation of transcription following oxidative stress likely depends on the regulatory activity of transcription factors such as SoxR, OxyR, PhoP, RpoS, or RpoE. We have clarified this point in the revised Discussion.

Our data indicate that the regulatory activity of Gre factors helps *Salmonella* apportioning energy generation between glycolysis and oxidative phosphorylation, thus eliciting metabolic signatures that promote resistance of *Salmonella* to oxidative stress. The importance of metabolism in the resistance of bacteria to oxidative stress is understudied. Therefore, our investigations on the transcriptional regulation of central metabolic pathways by the regulatory activity of Gre factors advance our understanding of the protective responses to oxidative stress.

The only control for specificity seems to be the DksA control.

Response: Our RNA seq analysis indicates that the majority of *Salmonella* genes are transcribed independently of the regulatory activity of Gre factors. The reviewer is correct that *dksA* gene is the only negative control used in our transcriptional pausing experiments. Specifically, our *in vitro* transcription assays have used of one negative control (i.e., *dksA*) for three positive targets (i.e., *gapA*, *eno* and *cydA*). Because transcriptional pausing is a quite time-consuming technique, we believe the use of *dksA* as control is justified.

It seems to make sense that oxidized nucleotides (or a decrease in nucleotide avaiability) might cause more aberrant and backtracking transcripts to form during elongation.  The fact that the pentose phosphate pathway is impacted also seems to suggest that nucleotides might be in short supply (or the cell THINKS they're in short supply)?  Although, of course, that pathway also controls the levels of NADPH used for most reductive/anabolic forms of metabolic pathways...so it's a complex question. But I'm simply not clear on why oxidative stress might increase the need for GreAB unless there's a corresponding increase in bad transcripts being formed during elongation.

- I guess I'm asking if an alternative model could be that oxidative stress drains metabolites from the pentose phosphate pathway away from nucleotide generation...this causes pausing during transcription...which needs GreAB to fix.

Response: When we started to look into the mechanisms by which Gre factors increase resistance of *Salmonella* to oxidative stress, we pondered the idea put forward by the reviewer. To test the rate of mutations in the transcriptome, we performed circular seq. However, the results of this approach were equivocal and we decided not to pursue this line of inquiry further. In response to the reviewer’s request, we have now analyzed RNA seq datasets for mutations in the transcriptome using state-of-the-art sequencing and bioinformatic approaches. As expected, *ΔgreAB Salmonella* grown in MOPS-GLC minimal medium contain more errors in the transcriptome than wildtype controls (**Fig. 2A**), likely influencing the poor growth of *ΔgreAB Salmonella* in glucose minimal media. Treatment of wildtype *Salmonella* with H_2_O_2_ increased the number of errors in the transcriptome, perhaps reflecting the misincorporation of nucleotides into the mRNA because of either their oxidation or because of changes in their proportion. Surprisingly, H_2_O_2_ treatment did not elevate the already high proportion of errors in the transcriptome of *ΔgreAB Salmonella* grown in glucose medium. However, compared to wildtype controls, *ΔgreAB Salmonella* undergoing peroxide stress seem to contain a greater number of transcriptional errors in genes associated with metabolism. Thus, the combined effects of decrease fidelity and inability of resolving pauses in ternary elongation complexes in metabolic genes may help explain the poor utilization of glucose by *ΔgreAB Salmonella* and the hypersusceptibilty of this strain to oxidative stress.

The RNA seq also showed that wildtype and *ΔgreAB Salmonella* similarly upregulate the expression of antioxidant defenses that rely on the detoxification or scavenging of ROS. Furthermore, the Δ*greAB* mutant seems to detoxify H_2_O_2_ with similar or even better kinetics than wildtype controls (**Fig. S6C**), suggesting that Δ*greAB* mutants are capable of producing functional catalase. Together, these investigations suggest that the hypersusceptibility of *ΔgreAB Salmonella* to oxidative killing cannot be explained by defects in the induction of detoxification or scavenging systems, but may depend on defective metabolic signatures that are associated with resistance to ROS. The revised manuscript has been edited accordingly.

Regarding the second question, Δ*greAB* *Salmonella* seem to flux higher carbon through the pentose phosphate pathway as suggested by their higher NADPH/NADP^+^ ratio compared to wildtype controls (**Fig. 4I**). As discussed in the revised manuscript, the increased utilization of the pentose phosphate pathway is not sufficient to protect *Salmonella* against oxidative stress without proper glucose utilization in glycolysis and the electron transport chain.

Because of our recent investigations on the role glycolysis plays in resistance of *Salmonella* to oxidative stress and because of the important role *gre* genes play in the growth *Salmonella* in glucose, the follow up investigations focused on understanding the mechanisms by which Gre factors control glucose utilization. This information has been clarified to the revised manuscript.

- given that over 1000 genes are differentially expressed between the wt and GreAB mutants it's hard to know where the 'specificity' is for these GreAB factors.  The levels of many of those transcripts could be regulated indirectly.

Response: We agree with the reviewer that many of the differentially expressed genes may be indirectly regulated by Gre factors. Our *in vitro* reconstituted systems demonstrate that Gre proteins directly activate transcription elongation of *gapA*, *eno* and *cydA*. The direct effects Gre proteins have on the transcription of these central metabolic genes may affect the energetics and redox balancing capabilities of *Salmonella*, thus impacting resistance of this bacterium to oxidative stress.

- In looking at where pausing is occurring in the three transcripts analyzed...is there any sequence motif or nucleotide bias that causes RNAP to stall in a Gre-dependent fashion?  Are these regions overrepresented in metabolic transcripts and absent in DksA?

Response: As the reviewer has commented above, the genes differentially regulated in *ΔgreAB Salmonella* and wildtype controls could reflect direct or indirect effects of Gre factors on transcription. To determine sequence motifs that require Gre-dependent function, other techniques such as Net seq would have been required. This is beyond the scope of the present work.

- If we grew GreAB cells anaerobically would their transcriptomes be closer to wild-type grown anaerobically as well (would the differences disappear without oxygen)?

Response: As requested by the reviewer, we have measured transcription of selected genes in wildtype and Δ*greAB* *Salmonella* grown in MOPS-glucose minimum medium under anaerobic conditions. As suspected by the reviewer, the expression of these genes is much closer between Δ*greAB* *Salmonella* and wildtype controls under anaerobic conditions (see graph below), providing a reasonable explanation for the similar growth patterns of anaerobic Δ*greAB* and wildtype *Salmonella*. That expression of genes encoding glycolytic and ETC functions is closer between Δ*greAB* and wildtype *Salmonella* grown under anaerobic conditions may reflect the fact that in Salmonella grow much slower under anaerobic compared to aerobic conditions. In order to present the manuscript in a clearer fashion, however, we have eliminated the data concerning anaerobic growth from the revised manuscript.

Later the paper shifts away from the central narrative of GreAB and tests a wide variety of metabolic pathways and their response to oxidative stress.  Some of the findings are less novel but the methylglyoxylate pathway was something I had not read about in this context and this data is quite intriguing.  However much of the data is followed up by speculation as to what is happening to the cell.  The extra explorations make more noise but don't really help us pin anything down to a clear model.

Response: Reviewers #2 and #3 have commented on the ramifications that the methylglyoxal pathway may have in the adaptation of *Salmonella* to oxidative stress. However, both reviewers suggested this work could be better presented in an independent paper. We can see the value of their suggestions, and have decided to eliminate the methylglyoxal data from the current manuscript.

The lack of certain amino acids is not well explained in terms of the larger model, for example.  Neither is a link to the stringent response.

Response: In order to simplify the manuscript, the metabolomic analysis of amino acids has been downplayed in the revised document. As suggested, we have tried to better convey that the activation of the stringent response in Δ*greAB* *Salmonella* reflects the nutritional stress of this mutant.

small things:
- the lower amounts of peroxides in GreAB cells isn't paradoxical if they express a lot more catalase (line 109 in text)

Response: The text has been modified following the reviewer’s comment.

- if the nuo/ndh mutants didn't grow on glucose (figure 7) then how was their ability to make peroxide assessed in figure 1?

Response: Indeed, the growth of the Δ*nuo*Δ*ndh* mutant is very slow in MOPS-GLC minimal medium. Production of H_2_O_2_ by Δ*nuo*Δ*ndh* was assessed after 12 h of growth, when the mutant reached an OD_600_ of ~0.25. This information has been clarified in the Material and Methods section. We have also observed that Δ*nuo*Δ*ndh* produce low concentrations of H_2_O_2_ when grown to log phase in LB broth (our unpublished observations).

- figure 7A...should grow in a control media without glucose (or with amino acids?) - are these growth defects specific to glucolytic growth or a general overall defect?

Response: As observed with Δ*greAB* *Salmonella*, the Δ*cydAB* strain used in our investigations grows normally in MOPS-CAA minimum medium, suggesting that Δ*cydAB* has growth defects for some but not all carbon sources. This information is now shown in **Fig. S6A**. Our new analyses also showed that Δ*nuo* Δ*ndh* and Δ*atpE* mutants fail to grow in MOPS-CAA medium. The failure of Δ*nuo* Δ*ndh* and Δ*atpE* mutants to grow in MOPS-CAA medium is consistent with our recent investigations that showed that the energetic output derived amino acid metabolism is highly dependent on the electron transport chain (PMID: 32133408).

So the paper starts out discussing stringent and GreAB and then veers a bit off that track and doesn't really settle in on a specific mechanism but rather tries to explain a broad range of disparate observations that don't always seem connected.  I'm sympathetic because these metabolism questions are very hard to get to the bottom of.  Very hard!  But with respect to GreAB I'm left wondering how much of this is simply a consequence of hitting a pleotropic set of factors and how much of this antioxidant activity is the real role of GreAB factors.

Response: Thanks for pointing out the difficulties of performing mechanistic research in metabolism. Our paper is consistent with the idea that central metabolism plays a critical role in resistance of *Salmonella* to oxidative stress. Gre factors enable the optimal allocation of resources between oxidative phosphorylation and glycolysis, a metabolic signature associated with resistance of *Salmonella* to ROS. Our data demonstrate that Gre factors increase the transcriptional fidelity of metabolic genes, and directly control transcription elongation of *gapA*, *eno*, and *cydA* genes. *Salmonella* mutants deficient in glycolytic and aerobic respiration demonstrate that these central metabolic pathways help *Salmonella* resist oxidative stress (reference 5 and present work). In our modest opinion, our complementary approaches provide mechanistic insights into how the regulation of transcription of central metabolic pathways contributes to the resistance of *Salmonella* to oxidative stress, an aspect little understood yet essential for the survival of this enteropathogen during exposure to ROS in the innate host response.


Responses to Reviewer #3:

The manuscript entitled: "Rescue of transcriptional pausing in metabolic genes jumpstarts Salmonella antioxidant defenses" by Kant et al describes experiments that attempt to link the Gre factors to metabolism that combats ROS stress in Salmonella. The manuscript is intriguing, but it suffers from a writing style that is confusing and the work has some serious limitations.

Response: We have tried to present the work more clearly.

A lot of space in the introduction is given to discussions of DksA, but it is not really important for this paper. Thus, it represents a distracting diversion. The clipped writing style also makes it difficult for appeal to a broad audience. The major rationale appears to be that Gre factors act similarly to DksA, so do they have a role in antioxidant defense? But what the authors actually establish is the role of Gre in metabolism.

Response: Our current investigations were motivated by a comment made by Dr. Michael Cashel regarding a recent paper from our lab (PMID: 30429329). Since both DksA and Gre factors bind to the secondary channel of RNA polymerase, he wondered if the latter may also help *Salmonella* withstand the toxicity of ROS. Given the structural similarities between these transcription factors, we feel the background on DksA is a good starting point for our investigations. Having said that, in response to the reviewer’s comment, we have shortened the introductory information on DksA and have expanded the background on Gre factors.

Over the years, we have studied the mechanism by which superoxide dismutases, glutathione, thioredoxin or SPI-2 gene products help *Salmonella* survive the antimicrobial activity of the phagocyte NADPH oxidase. More recently, we have discovered essential roles for glucose utilization in resistance of *Salmonella* to NOX2 killing, and our extensive work on the stringent response has revealed salient roles for metabolism in the antioxidant and anti-nitrosative defenses of *Salmonella*. Our investigations herein demonstrate important roles for Gre factors in the regulation of metabolic pathways associated with the resistance of *Salmonella* to oxidative stress. Thus, it is become apparent that metabolism is intimately associated with the resistance of microorganisms to oxidative stress.

As is the case for DksA, Gre factors may also play roles in the resistance of *Salmonella* to oxidative killing in ways that are independent of metabolism. For instance, the new analysis included in **Fig. 3** suggests that Gre factors directly or indirectly regulate SPI2 gene transcription, a locus associated with antioxidant defense. Gre factors also help *Salmonella* activate the expression of the Suf, as well as methionine and branch-chain amino acid biosynthesis genes. All of these loci have been associated with resistance to oxidative stress. The revised manuscript considers that the role Gre factors play in resistance of *Salmonella* to oxidative stress might not be limited to the regulation of transcription of genes encoding various steps of central metabolism, but may involve other loci as well.

This manuscript reads like a series of one-liners strung together. It is extremely disjointed. For example in the experiment shown in Fig. 1F, the authors state that greAB produced significantly more catalase activity without any explanation and suggest that the Gre factors resist oxidative stress in the innate host response by a mechanism that is independent from classical detoxification by catalases. They do not seem to explore this further but drop it out there without explaining what "classical" detoxification by catalases is for comparison. This type of writing is evident throughout the manuscript.

Response: We have majorly rearranged the information presented in the manuscript. We have also tried to improve the English.

Major criticisms are enumerated below.

1.      The authors immediately launch into an investigation of virulence in the mouse and the effect of eliminating greA, greB or both gre genes. A very difficult to follow section is then presented where the authors examine nos2-/- or nox2-/- mice. They provide too little rationale for the experiment and fail to state the significance of their findings. This part is written for a very specialized audience and not enough information is provided to the reader. It is clear that greAB is severely attenuated, also in the nox2 knockout, but not in the nos2 knockout. The authors start out with virulence in the mouse, rather than first establishing the effects of gre deletion in Salmonella. It is very sensitive to H2O2 stress (Fig 1C, D). In Fig. 1E, F the legends are woefully inadequate to describe the experiment.

Response: We normally focus our research efforts on loci with strong effects on the pathogenesis of *Salmonella*. Therefore, we first examined the virulence of Δ*greAB* *Salmonella* in C57BL/6 mice. Having established a major role for Gre factors in *Salmonella* pathogenesis, we then asked if the attenuation of Δ*greAB* *Salmonella* could be reversed in congenic mice deficient in the gp91*phox* subunit of the phagocyte NADPH oxidase. In response to the reviewer’s comments, we have tried to present the reason for using *nox2* and *nos2* mice in a clearer way. We have also revised the legends for easier interpretation of the figures.

2.      Then they move on to the effect of Gre and metabolism. It isn't clear what they mean by "grew poorly", because although in Fig. 1D the gre null strain has a slightly longer lag, it grows to higher density than the wt. Yet the authors do not comment on this. Why? Similarly in the presence of H2O2. In Fig. 2A anaerobic/GLC the authors need to extend the time points further to make their claim. Addition of GSH partially ameliorated the growth defect of the greAB null strain. Again, the writing style is a collection of lines centered around the figure rather than developing the narrative. STATE WHY THE ADDITION OF GSH IS SIGNIFICANT. The appropriate header for this section should be Gre factor mutants suffer from amino acid bradytrophy. ppGpp levels are up about 30%, is this enough to be functionally/ physiologically significant? The authors do not relate this finding back to the biology.
Response: We assayed growth of wildtype and a *ΔgreAB* mutant using both an automated plate reader and manual readings of bacteria grown in a shaker incubator. Both assays showed a prolonged lag phase for Δ*greAB* *Salmonella* when grown in glucose media compared to isogenic wildtype controls. However, only the mutants grown in the plate reader achieved higher densities than the wildtype controls. The arrangement of bacteria in the automated plates used to monitor bacterial growth can have an impact on the final OD. We have repeated the growth of wildtype and Δ*greAB* *Salmonella* in the plate reader., and have made a stronger effort to distribute the two strains at random in the multiwell plates. The new analyses indicate that wildtype and mutant grow to similar densities in a plate reader. The new data have been added to the revised **Fig. 1**. The analysis in **Fig. 1E** combines data from the old and new growth curves.

In order to simplify the paper, we have eliminated the experiments that tested the effects of GSH on the growth of Δ*greAB* *Salmonella*. For the same reasons, the experiments related to growth under anaerobiosis have been deleted from the revised manuscript.

We have not tested the functional consequences of the 30% increase in the concentration of ppGpp in Δ*greAB* *Salmonella*. We have used the changes in ppGpp content as an independent measure of nutritional stress. Having said that, slight changes in ppGpp content can have a significant impact on the transcriptional activity of RNA polymerase (see for example PMID: 30429329)

3.      In figure 3, the actual data are buried in the supplementary information (S3-4) and should be shown. It isn't clear what percentage of the overall transcriptome involves metabolism, since the authors only validated a subset of genes. But since they go on to further study the ones in the supplementary materials, the data should be in the main text. Why did the authors mention pykF, when it barely meets the threshold of 2 (and only because of an outlier)? The existing Figure 3 can be much smaller and moved to supplemental.

Response: The RNA seq data are presented in more detail in **Fig. 3** and **Fig. S2** of the revised manuscript. In response to the reviewer’s comments, we have eliminated the information related to *pykF*, and have erased the model originally presented in **Fig. 3**.

4.      A critical experiment that is missing here is an RNAseq analysis of the Salmonella response to ROS and how it compares to the greAB response. Does the methylgyoxal pathway show up? This would validate the findings of Fig. 6. Again, in this section the authors introduce another pathway and more components (gloB) without a proper introduction of the pathway. The authors conclude that formation of GSH through the cleavage of S-lactoyl-glutathione contributes to antioxidant defenses, but this is independent of greA/B (fig 6F), so why include here? Further, the authors then look at a role for gloB in virulence and include competitive infections, but do not thoroughly describe this figure. This seems like an aside, and it muddies the manuscript.

Response: As requested, we have added comparisons of RNA seq datasets from wildtype and Δ*greAB* *Salmonella* in response to oxidative stress (**Fig. 3B, 3E, S2**). Taking into account the comments made by Reviewers #2 and #3, we have eliminated the experiments related to the methylglyoxal pathway. This work will be presented in a separate paper.

5.      Then in the last part (Figure 7), the authors go back to redox, and this part is completely disconnected from greA/B. The authors do not connect the dots and it leaves the reader hanging and confused.

Response: We appreciate the reviewer’s comment and have segregated the information related to glycolysis and ETC in separate figures. We hope the rationale for the experiments presented in **Fig. 6** is more evident in the revised document.

6.      I think the authors should consider writing a paper that describes the role of Gre factors in Salmonella metabolism. Later work might be able to link it to ROS stress, but requires a lot more work to establish direct effects. Going back and forth between ROS, Gre, Metabolism and virulence is too confusing and is incomplete in its present form.

Response: As stated in other parts of the Response, an increasing body of evidence demonstrates an intimate link between metabolism and the ability of *Salmonella* to withstand oxidative stress. It is becoming evident that metabolism is thoroughly entangled in the resistance of bacteria to oxidative stress. We have tried to clarify how ROS, Gre factors, metabolism and virulence are intertwined.

Minor comments:

1. line 87 should be an increase in Salmonella fitness or increased Salmonella fitness

Response: As suggested, the change has been made.

2. Figure 1 is too crowded and the y-axes are so compressed that it is difficult to separate the strains in Fig. 1A especially.

Response: We have tried to present the data in **Fig. 1** more clearly. Hopefully, the increased quality of the figures will help the reader.

3. line 114 should be growth; line 115 should be grew

Response: The suggested changes have been made.

4. What was the pH of the growth media? I cannot find it mentioned in Figure legends or methods.

Response: The pH of the MOPS-GLC growth media is 7.2. This information has been added to the Methods and has been clarified in the legends.

5. there are lots of examples of poor English throughout, including supplementary figure legends. Line 145: "because of greAB Salmonella grow poorly" this doesn't make sense.

Response: The sentence has been revised according to the reviewer’s comment. We have tried to improve the English throughout the manuscript.

6. The figures are very difficult to read with small green text that fades into a gray background.

Response: As mentioned above in our response to Reviewer #1, we have tried to improve the quality of the figures. In addition, there should be links embedded in the Figures that access high resolution versions of the figures.
