# Supplementary material for: Gre factors help Salmonella adapt to oxidative stress by improving transcription elongation and fidelity of metabolic genes
Source: PLoS Biol. 2023 Apr 4;21(4):e3002051. doi: 10.1371/journal.pbio.3002051 (PMC10072461; doi:10.1371/journal.pbio.3002051)
Supplement: Attachment — Submitted filename: Response.docx [file pbio.3002051.s007.docx]

RESPONSE

ETHICS STATEMENT:

-- Please include the full name of the IACUC/ethics committee that reviewed and approved the animal care and use protocol/permit/project license. Please also include an approval number.
Response: The revised manuscript includes the Ethics Statement. We have also added the information regarding the IACUC/ethics committee and approval number.

-- Please include the specific national or international regulations/guidelines to which your animal care and use protocol adhered. Please note that institutional or accreditation organization guidelines (such as AAALAC) do not meet this requirement.
Response: All animals were handled in accordance with the Guide for the Care and Use of Laboratory Animals. This information has been clarified in the Ethics Statement.

2. Please provide a blurb which (if accepted) will be included in our weekly and monthly Electronic Table of Contents, sent out to readers of PLOS Biology, and may be used to promote your article in social media. The blurb should be about 30-40 words long and is subject to editorial changes. It should, without exaggeration, entice people to read your manuscript. It should not be redundant with the title and should not contain acronyms or abbreviations.

Response: Please, use the following blurb: “Control of transcriptional fidelity and elongation of electron transport and glycolytic genes by Gre factors ensures energy and redox balancing outcomes that help *Salmonella* thwart to the antimicrobial activity of phagocyte NADPH oxidase.”

3. We suggest a change in the title, but please modify as you consider necessary: "Gre factors help Salmonella adapt to reactive oxygen species (ROS) stress by improving transcription elongation and fidelity".

Response: As suggested, the title now reads “Gre factors help *Salmonella* adapt to oxidative stress by improving transcription elongation and fidelity of metabolic genes.”

As you address these items, please take this last chance to review your reference list to ensure that it is complete and correct.

Response: The references have been reviewed.

Reviewer #1: This new version is event better that the previous manuscript. I congratulate the authors for their excellent study.

Response: Thank you for your support.

Reviewer #2: This revision is a substantial improvement over the prior submission.  I think the incorporation of the RNAseq/transcript fidelity data and the streamlined presentation made it a much better read and a more complete story.  The authors ditched some data that was very distracting to the central theme and strengthened other aspects.  I believe this was the correct choice and that some of their other earlier findings probably belong in a separate publication.

Response: Thank you for the kind words.

I do think the story never really nails what GreAB is actually doing (which transcripts are targeted and why...signature mutations and motifs were not identifiable although I appreciate the authors looked and stuck to reporting the facts) and I do have some worries that most of these phenomena are side-effects of a highly pleiotropic mutant rather than direct evidence that there is a metabolic 'switch' controlled by GreAB that is activated in response to oxidative stress. That said there's a lot of novelty here and the experiments are pretty well controlled.  I believe the paper will be well read and cited and that this work is a valuable contribution to the field.

Response: We completely agree with the reviewer’s statement.

I have a few minor comments:

- given that peroxidases and catalases could account for consumption of H2O2, I think the assertion that 'catalase' activity is higher in GreAB mutant cells should be reworded.  (line 115)  Also I think they may be citing Fig 1F when they mean 1G?

Response: As indicated, in line 115 and elsewhere, we now use peroxidase rather than catalase. Having said that, the reported consumption of H_2_O_2_ most likely reflect the activity of catalases, because the disproportionation of this reactive species by catalases is kinetically more efficient than its consumption by peroxidases at the high concentrations of peroxide used in the assays.

- the extended lag phase in dicarboxylic acids like succinate has been described for salmonella by Hinton's group, Navarre's group (both groups in the past couple of years) and earlier work from Herb Shellhorn.  (line 161)  Probably should cite one of these.

Response: As suggested, we have cited one of these papers.

And some editorializing:

- line 25 = what is an 'emergent aspect'?  Keep it simple.

Response: The sentence has been modified.

- line 27 - is the stringent response (just) a 'metabolic program'?  It also shuts down ribosomal and tRNA synthesis.

Response: The reference to the stringent response now reads “Evolutionarily diverse bacteria combat the toxicity of reactive oxygen species by actively engaging the stringent response, a stress program that controls many metabolic pathways at the level of transcription initiation via guanosine tetraphosphate and the α-helical DksA protein.”

- Figure 6I where the authors show that the bd (cydAB) system is required for resistance to oxidative stress...seems very novel and important?  It barely gets a mention, whereas I think it should be in the abstract.

Response: As suggested, we have included the following sentence in the abstract: “In particular, the activation of cytochrome *bd* protects *Salmonella* from phagocyte NADPH oxidase-dependent killing by promoting glucose utilization, redox balancing and energy production.”


Reviewer #3: The manuscript "Transcription elongation and fidelity Gre factors jumpstart Salmonella oxidative stress resistance" by Kant et al is a revised manuscript. The manuscript has been substantially revised and the writing style has improved enormously. However, there are still some fundamental flaws as described below.

1.      The title should be changed, the authors never described how Gre "jumpstarts". The running title is more appropriate.

Response: The title has been changed to “Gre factors help *Salmonella* adapt to oxidative stress by improving transcription elongation and fidelity of metabolic genes.”

2.      Line 38 what is meant by a "pivotal breakpoint"? It isn't clear.

Response: The sentence in question now reads “Control of transcription fidelity and elongation by Gre factors represent important points in the regulation of metabolic programs supporting bacterial pathogenesis.”

3.      The bottom line here is that Gre factors help reprogram Salmonella metabolism in response to oxidative stress. The manuscript is largely a study of Salmonella metabolism and thus, it lacks broad general interest

Response: We respectfully disagree with this opinion. *Salmonella* is a model organism for which much of the paradigms in oxidative stress have been established. For instance, the OxyR regulon was first described in *Salmonella*. Gre factors are widely expressed by phylogenetically diverse organisms. Moreover, the generation of energy and balancing of redox in substrate-level phosphorylation or aerobic respiration are preserved in prokaryotic and eukaryotic organisms. The new understanding of the regulation of metabolic pathways by the regulatory activity of Gre factors during oxidative stress using *Salmonella* is likely generalizable to other organisms.

4.      L120 the header is completely uninformative. This exemplifies the problems with the writing style that make it difficult for a general audience. The header should be "the absence of gre factors does not exacerbate a decrease in transcriptional fidelity from peroxide stress" or something related. The reader is forced to wade through the entire paragraph to grasp the point. Similarly, and as a general comment, it is impossible to simply look at the figures and follow the thread, because the manuscript contains extraneous data that should be in supplementary and some data in supplementary should be in the main text.

Response: As suggested, we have modified the header to be more informative.

5.      Lines 137-139 the authors state: "The lack of substitution error type bias between untreated wildtype and greAB Salmonella conflicts with prior work in greAB E. coli that identified a strong G>A substitution bias [34, 35]. The authors suggest why this discrepancy might exist but do not examine experimentally.

Response: To get to the molecular mechanisms underlying the differences between Gre factors in *E. coli* and *Salmonella* are beyond the scope of these investigations.

6.      Lines 120-146 and Figure 2 are essentially negative data, as such, they detract and distract from the manuscript and should be moved to supplementary results. The conclusions: there is no specific SNS type that is inherently driving the overall transcription error rates in wildtype compared to greAB Salmonella, suggesting that in the absence of Gre factors, Salmonella experience nutritional shortages.

Response: Figure 2 shows that the basal level of transcriptional errors is higher in Δ*greAB* *Salmonella* than wildtype controls. Moreover, while the transcription error rate jumps in wildtype cells following exposure to H_2_O_2_, the high error in the transcripts of Δ*greAB* *Salmonella* undergoing peroxide stress does not change.

7.      Since the authors further study gapA, eno and cysA, Figure 3 should emphasize these specific genes and their expression in WT vs gre mutants in both ROS +/- conditions. Thus, in point 4 by reviewer 3 "A critical experiment that is missing here is an RNAseq analysis of the Salmonella response to ROS and how it compares to the greAB response" has not been fully addressed. The Table in supplemental should be moved to the main body.

Response: As requested by reviewers #2 and #3, the revised manuscript submitted in December included new RNA seq comparisons between H_2_O_2_-treated wildtype and H_2_O_2_-treated Δ*greAB* *Salmonella*. We prefer to show the data in question in a supplementary Table.

8.      Their observations suggests that Gre factors resolve transcriptional errors in transcripts encoding metabolic functions in Salmonella experiencing oxidative stress. The authors propose that Gre factors regulate assimilation of a variety of glycolytic sugars as well as various carbon sources that enter the TCA, allowing for the balanced production of amino acids. It is a pretty obvious conclusion.

Response: We agree with the reviewer that the transcriptional errors and prolonged pauses in metabolic genes recorded in Δ*greAB* *Salmonella* likely explain the growth defects and susceptibility to oxidative stress of this mutant strain.

9.      Differential expression analyses revealed changes in the transcription of 1,569 genes between greAB and wildtype Salmonella of which 944 were underexpressed and 623 were overexpressed in greAB Salmonella. Gre-dependent rescue of transcriptional pauses is an important step in the activation of key glycolytic genes in Salmonella. The major conclusion is that these investigations indicate that transcriptional control Gre factors exert on ETC genes fosters aerobic metabolism, thereby helping Salmonella meet their energetic and redox needs, while useful information, is hardly novel. The authors never get to the "How?"

Response: The results of the pausing experiments using *in vitro* transcription assays provide a logical explanation for the critical role Gre factors play in the expression of key glycolytic and ETC genes.
